# Deep CNN Model Using CT Radiomics Feature Mapping Recognizes EGFR Gene Mutation Status of Lung Adenocarcinoma

**DOI:** 10.3389/fonc.2020.598721

**Published:** 2021-02-12

**Authors:** Baihua Zhang, Shouliang Qi, Xiaohuan Pan, Chen Li, Yudong Yao, Wei Qian, Yubao Guan

**Affiliations:** ^1^ College of Medicine and Biological Information Engineering, Northeastern University, Shenyang, China; ^2^ Key Laboratory of Intelligent Computing in Medical Image, Ministry of Education, Northeastern University, Shenyang, China; ^3^ Department of Radiology, The First Affiliated Hospital of Guangzhou Medical University, Guangzhou, China; ^4^ Department of Electrical and Computer Engineering, Stevens Institute of Technology, Hoboken, NJ, United States; ^5^ Department of Electrical and Computer Engineering, University of Texas at El Paso, El Paso, TX, United States; ^6^ Department of Radiology, The Fifth Affiliated Hospital of Guangzhou Medical University, Guangzhou, China

**Keywords:** lung cancer, epidermal growth factor receptor mutation, deep learning, convolutional neural network, feature mapping

## Abstract

To recognize the epidermal growth factor receptor (EGFR) gene mutation status in lung adenocarcinoma (LADC) has become a prerequisite of deciding whether EGFR-tyrosine kinase inhibitor (EGFR-TKI) medicine can be used. Polymerase chain reaction assay or gene sequencing is for measuring EGFR status, however, the tissue samples by surgery or biopsy are required. We propose to develop deep learning models to recognize EGFR status by using radiomics features extracted from non-invasive CT images. Preoperative CT images, EGFR mutation status and clinical data have been collected in a cohort of 709 patients (the primary cohort) and an independent cohort of 205 patients. After 1,037 CT-based radiomics features are extracted from each lesion region, 784 discriminative features are selected for analysis and construct a feature mapping. One Squeeze-and-Excitation (SE) Convolutional Neural Network (SE-CNN) has been designed and trained to recognize EGFR status from the radiomics feature mapping. SE-CNN model is trained and validated by using 638 patients from the primary cohort, tested by using the rest 71 patients (the internal test cohort), and further tested by using the independent 205 patients (the external test cohort). Furthermore, SE-CNN model is compared with machine learning (ML) models using radiomics features, clinical features, and both features. EGFR(-) patients show the smaller age, higher odds of female, larger lesion volumes, and lower odds of subtype of acinar predominant adenocarcinoma (APA), compared with EGFR(+). The most discriminative features are for texture (614, 78.3%) and the features of first order of intensity (158, 20.1%) and the shape features (12, 1.5%) follow. SE-CNN model can recognize EGFR mutation status with an AUC of 0.910 and 0.841 for the internal and external test cohorts, respectively. It outperforms the CNN model without SE, the fine-tuned VGG16 and VGG19, three ML models, and the state-of-art models. Utilizing radiomics feature mapping extracted from non-invasive CT images, SE-CNN can precisely recognize EGFR mutation status of LADC patients. The proposed method combining radiomics features and deep leaning is superior to ML methods and can be expanded to other medical applications. The proposed SE-CNN model may help make decision on usage of EGFR-TKI medicine.

## Introduction

Lung adenocarcinoma (LADC) is a type of common lung cancer (1). Epidermal growth factor receptor tyrosine kinase inhibitor (EGFR-TKI) has become one significant target chemotherapy medicine for the treatment of the advanced LADC (2). To know the mutation status of EGFR gene in LADC patients is a prerequisite of deciding whether EGFR-TKI can be used (3). Polymerase chain reaction (PCR) assay or gene sequencing is the clinical method of measuring EGFR status, however, the tissue samples obtained by surgery or biopsy are required. The extensive intratumor heterogeneity may reduce the accuracy of EGFR gene measurement using the biopsy (4, 5). In addition, some patients may have inoperable LADC or the biopsy is not possible for the reason of patients’ endurance or willing or high economic cost. Therefore, it is necessary to find a non-invasive method to predict EGFR mutation status.

Computed tomography (CT) has been one non-invasive imaging technology and routinely used in cancer diagnosis and treatment (6, 7). Some studies have investigated the relationship between CT imaging features and EGFR mutation and provided the potential of using CT images to predict EGFR mutation status (8–10).

Radiomics aims to apply advanced computational approaches and artificial intelligence to convert medical images into quantitative features (11, 12). It has been utilized to help do the diagnosis and prediction of gene mutation, treatment response, and prognosis of lung cancer (13–15).

Recently, CT-based radiomics features and the resulted machine learning models have showed predictive value to EGFR mutation status. Dai et al. have trained one Random Forest (RF) model with 94 radiomics features, achieving an Area Under Curve (AUC) of 0.802 in a set of 345 patients (16). Zhang et al. have investigated 180 non-small cell lung cancer patients, extracted 485 features, and reached an AUC of 0.862 and 0.873 for the train and validation cohorts, respectively (17). Yang et al. have collected a total of 467 patients and created a predictive model which can recognize mutation status of EGFR gene with an AUC of 0.831 (18). More advanced methods of using radiomics features are required to improve the performance of EGFR mutation status prediction.

Deep Convolutional Neural Network (CNN) utilizes hierarchical network to learn abstract features and build up the mapping between input data and output labels. Deep learning has demonstrated excellent performance in many medical applications such as diagnosis of diabetic retinopathy (19), diagnosis of prostate cancer (20), differentiation of benign and malignant pulmonary nodules (21–23), classification of skin cancer (24), pediatric pneumonia diagnosis (25), and prediction of liver fibrosis (26). Moreover, deep learning models have been applied in lung cancer analysis (27–30). In EGFR mutation status, Wang et al. have constructed one deep learning model using 844 lung adenocarcinoma patients, which can achieve an AUC of 0.85 and 0.81 for the train and validation cohorts, respectively (31). Although some techniques such as deep dream and Grad-CAM (Gradient-weighted Class Activation Mapping) have been developed, the interpretation of the “black-box” of deep learning model still face challenges (32, 33).

In this work, we have proposed one new way of constructing a deep leaning model using CT radiomics feature mapping to precisely recognize EGFR mutation of lung adenocarcinoma. Specifically, for each LADC patient, after 1,037 CT-based radiomics features are extracted, 784 discriminative features are selected to be analyzed and construct a feature mapping. One Squeeze-and-Excitation (SE) Convolutional Neural Network (SE-CNN) is further designed and trained to recognize EGFR status from the radiomics feature mapping.

In summary, the contributions can be three aspects. First, the proposed method has utilized both the good interpretability of radiomics features obtained by feature engineering and the powerful capability of pattern recognition of deep learning. Second, the resulted SE-CNN model can precisely recognize EGFR mutation status from non-invasive CT images and the AUC can reach 0.910 and 0.841 in the internal and external test cohorts, respectively. It outperforms the CNN model without SE, the machine learning models, and the state-of-art models. Third, many discriminative features of imaging texture, intensity and the shape of lesion have been identified, which may help understanding the biological mechanism of EGFR mutation in lung LADC from the viewpoint of computer vision.

## Materials and Methods

### Study Design and Participants

This is one retrospective study and it has been approved by the ethics committee of The First Affiliated Hospital of Guangzhou Medical University and Shengjing Hospital of China Medical University. Patients who meet the following inclusion criteria are collected into this study: (a) the EGFR mutation status is examined by a PCR-based assay and confirmed by direct sequencing. The results of the EGFR test are clear; (b) All CT examinations are performed by the same CT scanner and with the same slice thickness and reconstruction algorithm; (c) Before receiving the CT examination, the patient has no extrathoracic metastasis and not received any radiotherapy or chemotherapy.

The exclusion criteria are given as follows: (a) The patient has received preoperative treatment or not been examined by the EGFR mutation test; (d) The clinical data of gender, age, and histopathological subtype is missing; (e) The radiomics features cannot be extracted accurately.

Totally a cohort of 709 patients (320 male and 389 female; the mean age of 59 years; the age range of 17–91 years) with LADC is included from The First Affiliated Hospital of Guangzhou Medical University. This cohort is named as the primary cohort in the following manuscript. These patients have been enrolled from January 2016 to July 2018. CT images and clinical data of all cases are collected. Clinical data collected from medical records for analysis includes EGFR mutation status, age, gender and histopathological subtype. LADC patients are divided into 8 different subtypes: acinar predominant adenocarcinoma (APA), micropapillary predominant adenocarcinoma (MPA), lepidic predominant adenocarcinoma (LPA), papillary predominant adenocarcinoma (PPA), solid predominant adenocarcinoma (SPA), invasive mucinous adenocarcinoma (IMA), minimally invasive adenocarcinoma (MIA), and adenocarcinoma in situ (AIS).

A cohort of 205 patients (101 male and 105 female; the mean age of 60.7 years; the age range of 32–88 years) with LADC is included from Shengjing Hospital of China Medical University. It is named as the external test cohort. It is noted that the data of histopathological subtype is lack in this cohort.

### Measurement of EGFR Mutation Status

After being fixed with formalin, the excised specimen is stained with H&E. Experienced pathologists evaluate the paraffin specimens of the LADC tissue and confirm that they contain at least 50% tumor cells. According to strict protocol from manufacturers, EGFR status is examined by a PCR-based assay and confirmed by direct sequencing. The status of EGFR exons 18, 19, 20, and 21 is also examined by molecular analysis.

### Acquisition of CT Images

For the primary cohort, a multi-detector CT system with 128 slices (Definition AS+, Siemens Healthcare, Germany) has been applied for the chest scans. All images are stored and exported in the format of DICOM. The parameters used in the CT examination are given as follows: The tube current modulation is 35–90 mAs; the tube voltage is 120 kVp; the spacing is 0.625 mm×0.625 mm; the reconstruction thickness is 2.00 mm; the matrix is 512×512; the field of view is 180 mm×180 mm; the reconstructed algorithm is B30 or I30; and the pitch is 0.9.

For the external test cohort, four CT scanner from different manufacturers (GE Medical Systems, Philips, Siemens and Toshiba) have been used for the chest scans. The pixel spacing ranges from 0.625 to 0.976 mm, the slice thickness ranges from 2.50 to 5.0 0 mm, and the matrix is 512×512.

### Overview of the Study Procedure

**Figure 1:**
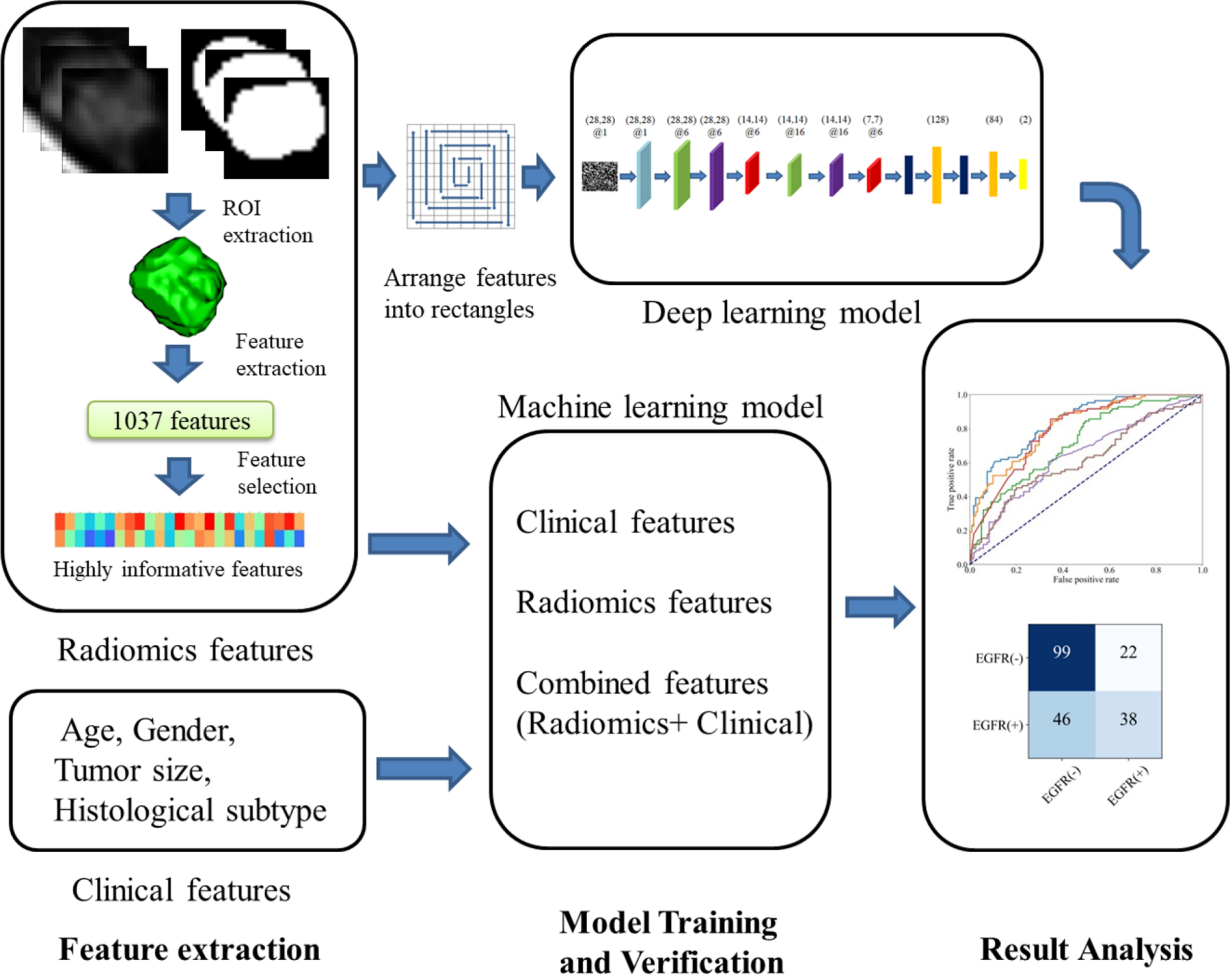
Flow chart of our study.

As given in **Figure 1**, for each LADC patient, after extracting 1,037 radiomics features from the segmented lesion region, 784 highly informative features are selected to be analyzed and construct a feature mapping. Convolutional Neural Network with Squeeze-and-Excitation (SE-CNN) is designed and trained to recognize EGFR status from the radiomics feature mapping. For comparison, CNN model without Squeeze-and-Excitation (SE), 1D-CNN, and the machine learning (ML) models using radiomics features, clinical features, and both features have also been implemented. Meanwhile, the highly informative features are analyzed. All models are trained and validated by using 638 patients from the primary cohort and tested by using the rest 71 patients (the internal test cohort). Furthermore, the models are evaluated by using the external test cohort of 205 patients.

### Extraction and Selection of Radiomics Features

We have used a 3D U-Net model for the nodule segmentation, which has been presented and used in our previous study (34, 35). After automatic segmentation, one radiologist with more than 10 years of experience in interpretation of lung CT images has checked the quality of each case and manually revised a few cases with poor tumor contours. And then PyRadiomics software (https://pyradiomics.readthedocs.io/) is utilized to extract features (36). A total of 1,037 radiomics features are extracted for each patient. The radiomics features can be divided into six different groups: Shape Features, First Order Features, Gray Level Co-occurrence Matrix (GLCM) Features, Gray Level Dependence Matrix (GLDM) Features, Gray Level Run Length Matrix (GLRLM) Features and Gray Level Size Zone Matrix (GLSZM) Features. Those radiomics features are extracted from three types of images: Original Image, Wavelet Image and Laplacian of Gaussian (LoG) Image. Wavelet Image is obtained from eight decompositions after wavelet filtering. Applying High (H) or Low (L) pass filter in three dimensions gives eight kinds of combinations: LHL, HHL, HLL, HHH, HLH, LHH, LLH, and LLL. LoG Image is generated through applying a LoG filter with a specified sigma value to the input image. It emphasizes the area where the gray scale changes. In LoG images, a low sigma emphasizes fine textures and a high sigma emphasizes coarse textures. Sigma of 1, 2, and 3 has been used in our study, respectively.

We have used the mean decrease impurity importance to reduce redundant radiomics features, which derived from the random forest (RF) method (37). Each radiomics feature is given an importance score in mean decrease impurity importance method. The purpose of feature selection is to identify highly discriminative features and remove unimportant or irrelevant radiomics features. It is noted that the feature selection is based on the training and validation cohort (638 of 709 patients), not the whole primary cohort.

### SE-CNN Model Using Radiomics Feature Mapping

**Figure 2:**
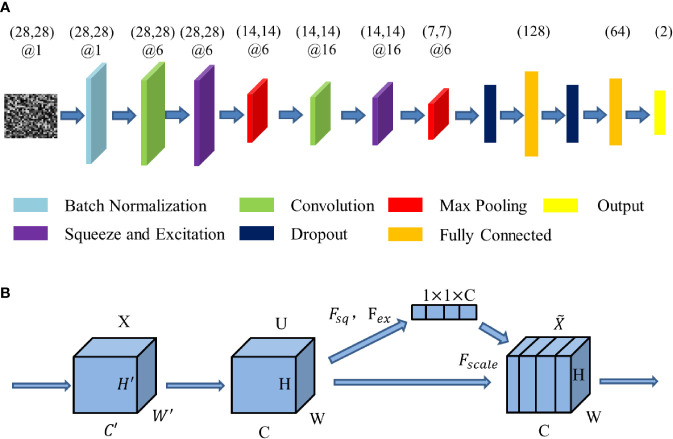
The structure of the deep learning model. **(A)** The structure of SE-CNN. **(B)** The structure of Squeeze-and-Excitation (SE) layer.

We have built a SE-CNN classifier with radiomics feature mapping as inputs. The structure of proposed SE-CNN model is presented in **Figure 2A**. It consists of the convolution layer, pooling layer, Squeeze-and-Excitation (SE) layer, dropout layer, and full connection layer.

Squeeze-and-Excitation (SE) layer can be tread as a channel’s self-attention function intrinsically. SE layer recalibrates channel-wise feature responses and learns the global information through suppressing less useful features and emphasizing informative features. Meanwhile, the benefit of the feature recalibration can be accumulated through SE layers. It has been proved that SE layer can improve CNN’s performance (38). The structure of the SE layer is shown in **Figure 2B**.

Squeeze-and-Excitation layer is special calculation unit. Input X has been transformed into feature U at first. Here * denotes convolution. Every Squeeze-and-Excitation layer is special calculation unit. Input *X* has been transformed into feature U at first. Here * denotes convolution. Every $v_{c}^{s}$ is a single channel of $v_{c}=\left[v_{c}^{1},v_{c}^{2},\ldots v_{c}^{C\prime }\right]$. These spatial kernels are applied to the relevant channel of *X*.

(1) $${\text{u}}_{c}=v_{c}*X=\Sigma_{a=1}^{C\prime }v_{c}^{s}*X^{s}$$

*F_sq_* generates statistics z by shrinking the spatial size of the global spatial information U = [*u*_1_,*u*_2_,...,*u_c_*] through its spatial dimensions *H* × *W* and squeezing it into the channel descriptor.

(2) $${\text{z}}_{c}=F_{sq}\left(u_{c}\right)=\frac{1}{H\times W}\Sigma_{i=1}^{H}\Sigma_{j=1}^{W}u_{c}(i,j)$$

The function of F_ex_ is to capture the channel dependencies. Its purpose is to fully utilize the information summarized in the squeeze operation. σ indicates the ReLU function.

(3) $$\text{s}={\text{F}}_{ex}(z,W)=\sigma \left(g(z,W)\right)=\sigma (W_{2}\delta (W_{1}z))$$

By activating the rescaling U, the output can be gotten as a block $\tilde{\text{X}}$.

(4) $${\tilde{x}}_{c}=F_{scale}(u_{c},s_{c})=s_{c}u_{c}$$

where $\tilde{\text{X}}=[{\tilde{x}}_{1},{\tilde{x}}_{2},\ldots ,{\tilde{x}}_{c}]$ and *F_scale_* indicates that the feature map *u_c_* is multiplied by the scalar *s_c_* at the channel-wise.

Since our study is a binary classification, the loss function of binary cross-entropy is employed in deep learning models.

(5) $$\text{loss}=-\Sigma_{i=1}^{n}{\hat{y}}_{i}logy_{i}+(1-{\hat{y}}_{i})\text{log}\left(1-y_{i}\right)$$

(6) $$\frac{\partial \text{loss}}{\partial \text{x}}=-\Sigma_{i=1}^{n}\frac{{\hat{y}}_{i}}{y_{i}}-\frac{1-{\hat{y}}_{i}}{1-y_{i}}$$

In the formula, *ŷ_i_* is the true label and *y_i_* is the label predicted by the model.

### The Training and Evaluation of the Deep CNN Models

First, we rank the 784 radiomics features with high scores in feature selection into a two-dimensional matrix. The arrangement rule is that the feature with higher score is near the center and that with lower score is near the edge. The arranged matrix of 28×28 pixels is treated as a feature mapping. After batch normalization, each two-dimensional feature mapping is input into the SE-CNN model as an image for training. By activating different SE, convolution and pooling layers, the SE-CNN classifier gives an EGFR-mutant probability for each patient.

In order to verify the function of SE layer, we have also established a deep learning model (CNN) without a SE layer. The CNN model has the architecture as same as that of SE-CNN model removing two SE layers. Due to the limited number of cases, this CNN model only has two convolutional layers. In our previous study, we have found that this kind of agile CNN is very suitable for a small dataset and images with a small size (39). Moreover, one 1D-CNN model with 2 convolutional layer, 1 max pooling layer and 1 average pooling layer (Please refer to **Supplementary Figure 1** to know the detailed architecture and parameter settings) is constructed and trained by the 1D vector of features.

To further confirm this point, we have done another three comparative experiments. The first one is AlexNet with five convolutional layers and it is trained from the scratch. The second and third are the pre-trained VGG-16 and VGG-19 with fine tuning, respectively (40). Specifically, all parameters in convolution layers except the final fully-connected layer are initialized using VGG-16 and VGG-19 trained by ImageNet dataset of 1.28 million natural images and fine-tuned by our own images. The final fully-connected layer is trained only using our own images. This kind of scheme has been proved to be one powerful way of using deep CNNs in medical imaging applications (31, 41).

For the training of SE-CNN, CNN, 1D-CNN, AlexNet, VGG16, and VGG19, the batch size and learning rate are set as 50 and 0.001, respectively. Binary cross-entropy loss function and adaptive moment estimation optimizer are used in SE-CNN, CNN, 1D-CNN, and AlexNet. Categorical cross-entropy loss function and RMSprop optimizer are adopted in VGG16 and VGG19. In our training, due to the small number of samples, to avoid over-fitting, we have used an early stopping method. When the validation loss does not drop for 5 consecutive epochs, the training stops. After continuous debugging, the model performance is verified by the ROC curve, AUC, accuracy, recall, and precision.

### Machine Learning Models for Comparison

For comparison, we have trained five machine learning models with different features and classifiers for EGFR mutation prediction. Using radiomics features, we train three machine learning classifiers, i.e., Random Forest (RF), Support Vector Machine (SVM), and Multilayer Perceptron (MLP). These classifiers have been shown to perform well in lung imaging analysis (21). Using the clinical information of gender, histopathological subtype and age as features, we have trained one machine learning model (SVM). Finally we have built a combined model (SVM) which using radiomics features and clinical information.

In SVM, C and gamma is set as 3 and 1, respectively. In RF model, four estimators are included. In MLP, two hidden layers with size of 10 and 5 are included and ReLu activation function and ADAM optimizer have been used.

### Software Tools and Experimental Environments

All statistical analyses have been done by using Python 3.6. The scikit-learn package is used to construct all machine learning models using radiomics features, clinical features, and both features. The implementation of the deep learning models (SE-CNN and CNN) is done by the Keras toolkit. Meanwhile, “matplotlib” package is employed to plot the ROC curves and data distribution. The independent two-sample t-test is adopted to evaluate the difference of age and classifier score between EGFR-positive [EGFR(+)] and EGFR-negative [EGFR (<x></x>–<x></x>)] groups. When a two-sided p-value is <0.05, it is considered to be significant. All experiments have been performed using a HPZ840 workstation, where the CPU and GPU are Intel Xenon E5-2640 v4 @ 2.40 GHz and Quadro M4000, respectively.

## Results

### Demographic and Clinical Characteristics

As shown in **Table 1**, for the primary cohort, EGFR(+) and EGFR(-) groups have shown no significant difference in age (p = 0.034), but shown significant difference in tumor size, gender, and subtype (p < 0.01). The mean age of EGFR(+) is 60.24 and the mean of EGFR(-) is 58.57. EGFR(+) has significantly higher proportion in men (64.7%) than in women (37.9%) (p < 0.01). In different subtypes, the number of APA is the largest in both EGFR(+) and EGFR(-), reaching 208 cases and 96 cases, respectively. The smallest subtypes in EGFR(+) are AIS and IMA, with only five cases. The least number among the EGFR(-) is the 15 cases of LPA. There is significant difference of EGFR(+) percentage between different subtypes of LADC.

**Table 1:** Demographic and clinical characteristics of LADC patients.

Characteristics		EGFR(-)	EGFR(+)	*p* value
***The primary cohort***				
Number of patients		352	357	
Age, mean		58.57	60.24	0.034
Gender	Male	114	206	< 0.01
	Female	238	151	
Tumor size (mean, mm^3^)		19,116	12,143	< 0.01
LADC subtype	AIS	17	5	< 0.01
	MIA	45	27	
	LPA	15	63	
	APA	96	208	
	PPA	66	51	
	MPA	21	15	
	SPA	49	8	
	IMA	18	5	
***The external test cohort***				
Number of patients		121	84	
Age, mean		61.07	59.26	< 0.01
Gender	Male	72	29	0.18
	Female	49	55	
Tumor size (mean, mm^3^)		26,598	17,926	< 0.01

LADC, Lung Adenocarcinoma; EGFR, Epidermal Growth Factor Receptor; AIS, Adenocarcinoma In Situ; MIA, Minimally Invasive Adenocarcinoma; LPA, Lepidic Predominant Adenocarcinoma; APA, Acinar Predominant Adenocarcinoma; PPA, Papillary Predominant Adenocarcinoma; MPA, Micropapillary Predominant Adenocarcinoma; SPA, Solid Predominant Adenocarcinoma; IMA, Invasive Mucinous Adenocarcinoma.

For the external test cohort, EGFR(+) and EGFR(-) groups have shown no significant difference in gender or CT scanner (p = 0.18; p = 0.17), but shown significant difference in age and tumor size (p < 0.01).

### Analysis of Predictive Radiomics Features

**Figure 3:**
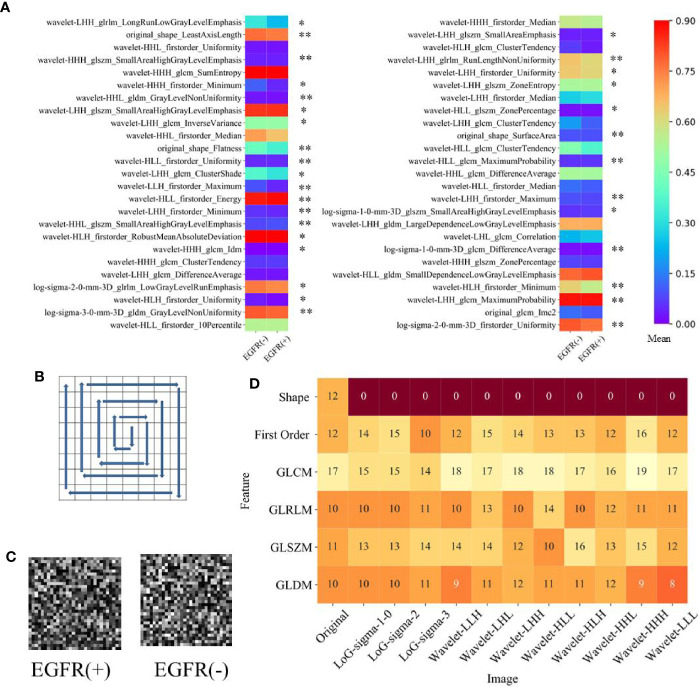
The selected radiomics features. **(A)** The mean value of 50 highly informative features in EGFR(+) and EGFR(-). *represents p <0.05 between EGFR(+) and EGFR(-), **represents p <0.001 between EGFR(+) and EGFR(-). **(B)** Clockwise sorting method for input matrix of deep learning model. **(C)** The example of the input matrix. **(D)** Distribution of selected 784 features used in deep learning models.

Fifty highly informative features have been selected to build the machine learning models (RF, SVM, MLP). The number of features is determined by the rule of thumb, i.e., each feature corresponds to 10 samples (patients) in a binary classifier (7). The 50 selected highly informative radiomics features are shown in **Figure 3A**, and clinical features are listed in **Table 1**. Among these 50 features, the features of First Order Features have the largest number, reaching 17. Meanwhile, 15 radiomics features are from Wavelet_LHH images, and 8 radiomics features are from Wavelet_HLL images.

Using an independent two-sample t-test on the datasets, we have compared the intensity of features between EGFR(+) and EGFR(-) groups. Among 1,037 feature, all 12 features of Short Run Emphasis in EGFR and 9 of 12 features of Sum Entropy are higher (overrepresented) in EGFR(+), indicating higher intratumor heterogeneity. For inverse variance quantifying homogeneity, 7 of 12 features are lower in EGFR(+) group. The mean value of the top 50 high-scoring features on EGFR(+) and EGFR(-) groups is shown in **Figure 3A**. Among these 50 features, there are 32 features with significant difference between EGFR(+) and EGFR(-) groups. For EGFR(+) group, the Minimum and DifferenceAverage features are higher than EGFR(-) group, but the radiomics features of Uniformity are lower in the EGFR(+) group. The largest difference between the mean values of EGFR (+) and EGFR (-) is wavelet-HLL_glcm_MaximumProbability feature (0.366 vs 0.458). There are 34 radiomics features that EGFR(-) is greater than EGFR(+), and only 16 EGFR(+) are numerically greater than EGFR(-).

Since the input of a deep learning network is a feature mapping of 28×28, we select 784 features with high score and rearrange them. We use counterclockwise to arrange the features into squares. The process is shown in **Figure 3B**. Meanwhile, the example of an arranged matrix on EGFR(+) and EGFR(-) is shown in **Figure 3C**. **Figure 3D** shows the distribution of the selected 784 radiomics features.

In **Figure 3D**, we have found that the number of GLCM features is the largest. Analyzed by proportion, 77.4% of the shape features and 81.7% of the GLSZM features are selected from 1,037 features and added to 784 features used by the deep learning models. The most features are for texture (614, 78.3%) and the features of the first order of intensity (158, 20.1%) and the shape features (12, 1.5%) follow. It is noted that the analysis in this section is based on the primary cohort.

### Performance of Deep Learning Models Using Radiomics Feature Mapping

**Figure 4:**
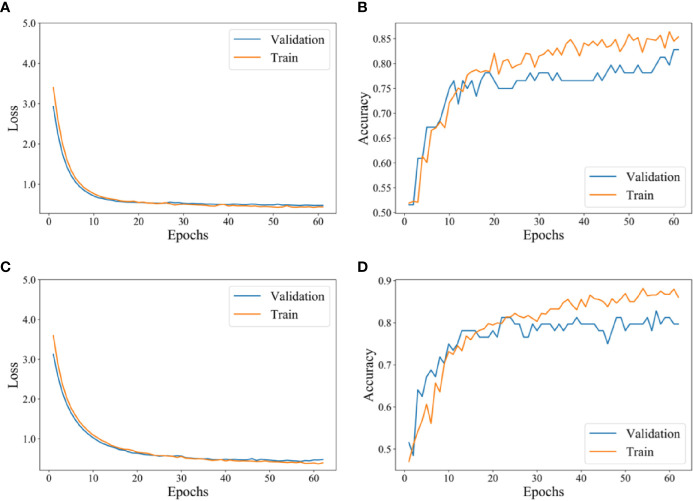
The training process of the deep learning model. **(A)** Loss curve of SE-CNN with epoch. **(B)** Accuracy curve of SE-CNN with epoch. **(C)** Loss curve of CNN with epoch. **(D)** Accuracy curve of CNN with epoch.

**Figure 4** shows the training process of deep learning models (SE-CNN and CNN). In the loss curve on **Figures 4A** and **C**, we can see that the curve of loss tends to be flat in SE-CNN and CNN models as the epochs number increases, indicating that the training model converges. In the accuracy curve on **Figures 4B** and **D**, the value fluctuates greatly, which may be because the number of training epoch is relatively small. SE-CNN model stops training early when the epoch is 61, while the epochs in the CNN model are 63. SE-CNN model reaches the convergence faster than CNN model and the final accuracy is also significantly higher.

**Figure 5:**
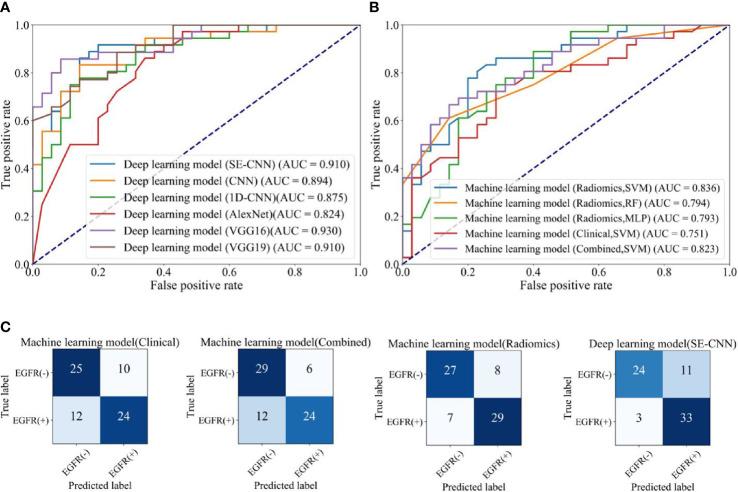
The EGFR status recognition performance of different models for the internal test cohort (71 of 709 patients). **(A)** ROC curves of different deep learning models. **(B)** ROC curves of machine learning models using clinical features, radiomics features (SVM, RF, MLP), and combined features. **(C)** Confusion matrix of machine learning model using clinical features, machine model using combined features, machine learning models (SVM) using radiomics features, and deep learning model (SE-CNN).

For the internal test cohort of 71 patients, the performance of deep learning models (SE-CNN and CNN) has been given in **Table 2** and **Figure 5**. The AUC of proposed SE-CNN is higher than that of CNN model (0.910 versus 0.894). 1D-CNN can achieve an AUC of 0.875 lower than that of SE-CNN. Moreover, though the AlexNet trained from the scratch has deeper architecture than our specifically designed SE-CNN and CNN, its performance is comparable to our models. Using fine tuning, the deeper VGG16 and VGG19 obtain the better prediction performance than AlexNet. Especially, the fine-tuned VGG19 achieve an AUC of 0.929.

**Table 2:** Predictive performance of machine learning models using radiomics features (SVM, RF, MLP), clinical features, and combined features, and the deep learning models (SE-CNN CNN, AlexNet, Fine-tuned VGG16, and Fine-tuned VGG19) for the internal test cohort of 71 patients.

Model	Feature	Classifier	Accuracy	AUC	Recall	Precision	F-score
Machine learning	Radiomics	SVM	0.788	0.836	0.805	0.784	0.794
		RF	0.732	0.794	0.611	0.814	0.698
		MLP	0.746	0.793	0.888	0.695	0.780
	Clinical	SVM	0.690	0.751	0.666	0.705	0.685
	Combined	SVM	0.746	0.823	0.666	0.8	0.727
Deep learning	Radiomics	**SE-CNN**	0.803	**0.910**	0.916	0.75	0.825
		CNN	0.816	0.894	0.833	0.812	0.821
		1D-CNN	0.760	0.875	0.833	0.731	0.779
		AlexNet	0.676	0.824	0.972	0.614	0.752
		Fine-tuned VGG16	0.828	0.930	0.714	0.925	0.806
		Fine-tuned VGG19	0.728	0.910	1	0.648	0.786

The meaning of the bold values is the model with the highest AUC.

**Figure 6:**
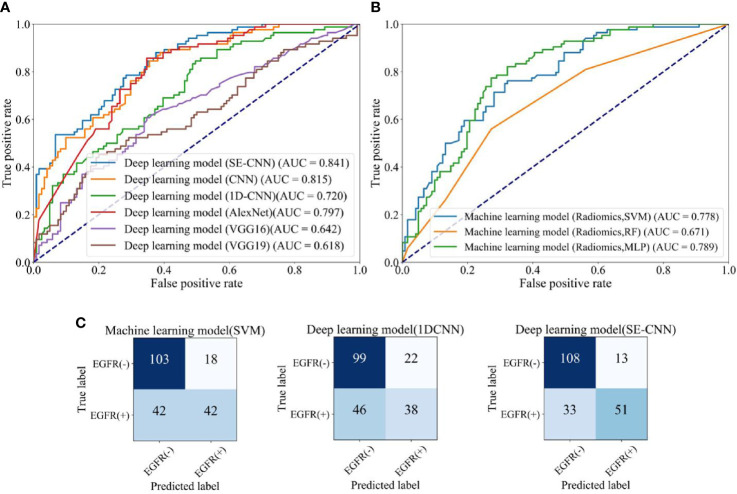
The EGFR status recognition performance of different models for the external test cohort (205 patients). **(A)** ROC curves of different deep learning models. **(B)** ROC curves of machine learning models (SVM, RF, MLP). **(C)** Confusion matrix of machine learning model (SVM) using radiomics features, 1D-CNN, and SE-CNN.

As shown in **Table 3** and **Figure 6**, all six deep learning models have lower AUC in the external test cohort than in the internal test cohort. The possible reason might be that the radiomics features and the resulted machine learning models have been influenced by the differences between different CT scanners, protocols, and hospitals (6). SE-CNN has the highest AUC of 0.841 among the six deep learning models. The AUC of Fine-tuned VGG16 decreases dramatically from 0.929 (the internal test cohort) to 0.642 and the AUC of Fine-tuned VGG19 decreases from 0.909 (the internal test cohort) to 0.618.

**Table 3:** Predictive performance of machine learning models using radiomics features (SVM, RF, MLP), and the deep learning models (SE-CNN CNN, AlexNet, Fine-tuned VGG16, and Fine-tuned VGG19) for the external test cohort of 205 patients.

Model	Classifier	Accuracy	AUC	Recall	Precision	F-score
Machine learning	SVM	0.707	0.778	0.500	0.700	0.583
	RF	0.619	0.671	0.261	0.579	0.361
	MLP	0.702	0.789	0.571	0.657	0.611
Deep learning	**SE-CNN**	**0.775**	**0.841**	**0.607**	**0.796**	**0.689**
	CNN	0.726	0.815	0.595	0.694	0.641
	1D-CNN	0.668	0.720	0.452	0.633	0.528
	AlexNet	0.688	0.797	0.904	0.575	0.704
	Fine-tuned VGG16	0.644	0.642	0.345	0.617	0.442
	Fine-tuned VGG19	0.549	0.618	0.607	0.463	0.525

The meaning of the bold values is the model with the best performance for the external test cohort.

### Comparison with Machine Learning Models

For the internal test cohort of 71 patients, the five machine learning models’ performance is listed in **Table 2**. The ROC curves and AUC are depicted in **Figure 5**. The clinical model using SVM does not have good prediction and its AUC is only 0.751. In the three machine learning models using radiomics features, the SVM model has obtained the best performance and the AUC reaches 0.836. Therefore, we use SVM to build the combined model and it has an AUC of 0.823. F-score in SVM models using radiomics features and combined features is 0.794 and 0.727, respectively.

As shown in **Figure 5A**, SE-CNN has better predictive performance than the clinical model (AUC: 0.910 versus 0.751). For the three models using radiomics features, SVM model is the best and RF and MLP models follow (AUC: 0.836, 0.794, and 0.793, respectively). Comparing **Figures 5A** and **B**, one can find that deep learning models of SE-CNN, fine-tuned VGG16 and VGG19, and CNN outperform the machine learning models. Even the CNN with two layer convolutional layers trained from the scratch has the higher AUC of 0.894 than that of the best machine learning model of SVM (0.836).

The confusion matrices of SE-CNN model and the three machine learning models (Clinical, Combined, and Radiomics) are shown in **Figure 5C**. We find that compared with three machine learning models, SE-CNN model has an improvement in the ability of predicting EGFR(+). Compared with the machine learning model (Radiomics + SVM), SE-CNN model has increased four correctly predicted cases in EGFR(+).

For the external test cohort, the comparison between SE-CNN and machine learning models are given in **Table 3** and **Figure 6**. SE-CNN achives an AUC of 0.841, higher than that of SVM, RF, and MLP models (AUC: 0.778, 0.671, 0.789). As shown in **Figure 6C**, SE-CNN model has an improvement in the ability of predicting both EGFR(+) and EGFR(-). Compared with the machine learning model (Radiomics + SVM), SE-CNN model has increased nine and five correctly predicted cases in EGFR(+) and EGFR(-), respectively. Meanwhile, compared with the deep learning model (1D-CNN), SE-CNN model has increased 13 and 9 correctly predicted cases in EGFR(+) and EGFR(-), respectively.

### Comparison with Available State-of-Art Models

**Table 4** summarizes some recently conducted works on EGFR mutation status. By using manually extracted image features and radiomics method, Velazquez et al. have obtained an AUC of 0.69 for a dataset of 353 patients (10). Gevaert et al. have achieved an AUC of 0.89, but their dataset only includes 186 patients (42). In the study done by Liu et al. using a logistic regression model, the AUC can reach 0.766 and 0.748 for the train and validation cohorts, respectively (13). Yang et al. have also achieved a good performance by RF model (18). By using deep learning method, Wang et al. have gotten encouraging predictive performance (AUC = 0.85) in a large dataset which has 800 patients (31). Compared with these results, our SE-CNN model has presented comparable prediction in EGFR mutations status.

**Table 4:** Performance comparison between our EGFR predictive model and the state-of-art.

Experiments	Years	Method	Number of Patient	AUC
Gevaert et al.	2017	Decision tree	186	0.89
Liu et al.	2017	Logistic regression	170	0.766
Velazquez et al.	2018	Deep belief network (DBNs)	353	0.69
Wang et al.	2019	Deep Learning(CNN)	800	0.85
Yang et al.	2019	RF model	467	0.831
Our method	2020	SE-CNN + Radiomics mapping	71/709; 205	0.910; 0.841

AUC, Area Under Curve; RF, Random Forest; SE, Squeeze-and-Excitation layer; CNN, Convolutional Neural Network.

### CT Images of Typical Examples of Recognition Results

**Figure 7:**
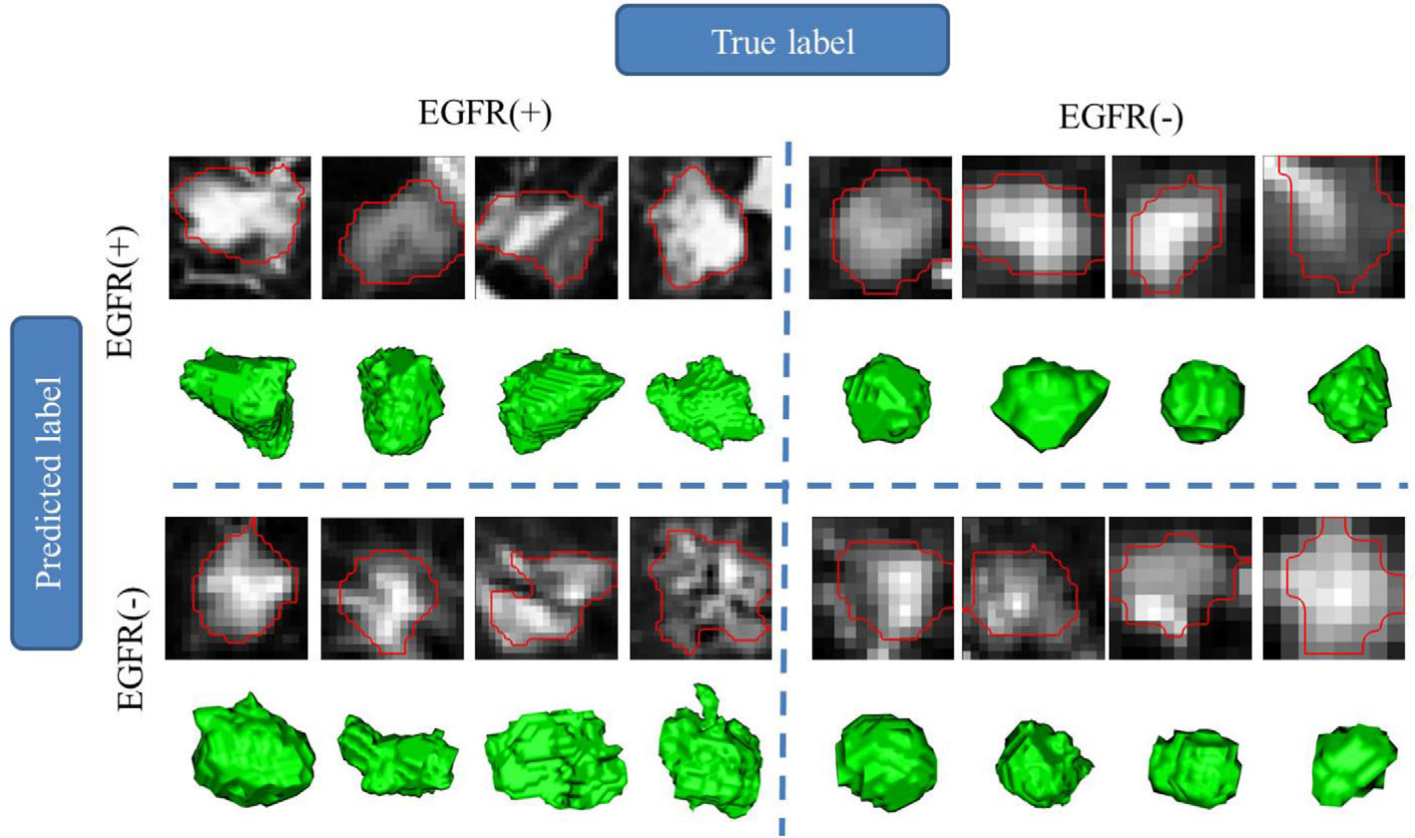
Region of interest (lesion) and 3D visualization for different EGFR status prediction in examples.

To demonstrate our results in one visible way, **Figure 7** gives some randomly chosen examples. The randomly selected images are divided into four parts, the predicted label is the same as the real label in EGFR(+) and EGFR(-), and the predicted label is different from the real label in EGFR(+) and EGFR(-). For each tumor lesion, one representative 2D patch with marked contour and 3D visualization are shown in **Figure 7**. From the 3D visualization, we have found that the nodule shape of EGFR(+) is relatively irregular, the lesion margin has microlobulated, angular and speculated. Meanwhile, the shape of the nodule EGFR(-) is relatively regular and the surface is relatively smooth. The shape of the lesion is closer to a sphere or ellipsoid in EGFR(-) cases. In 2D CT patch, if the contour of the lesion is relatively smooth and the internal texture is uniform, it is likely to be predicted as EGFR(-). Conversely, if the lesion contour is sharp and angular and the texture is turbid and complex, it is easy to be predicted as EGFR(+).

## Discussions

In this study, we have studied the relationship between the radiomics phenotype and the genotype of EGFR mutation in LADC. The clinical, imaging, and EGFR mutational profiling data of 709 LADC has been analyzed. One new method of using the deep CNNs and CT-based radiomics feature mapping has been proposed to predict EGFR mutation status. It is found that EGFR(-) patients show the smaller age, higher odds of female, larger lesion volumes, and lower odds of subtype of APA. The most discriminative features are intratumor heterogeneity in the form of texture. The resulted SE-CNN model can recognize EGFR mutation status with an AUC of 0.910 and 0.841 for the internal and external test cohorts, outperforming the CNN model without SE, three ML models and the state-of-art models.

### Predictive Radiomics Features of EGFR Mutation Status

In this study, the mean decrease impurity importance method has been to select predictive features. This method has been utilized in our previous study (18). Among the selected features, the most features are for texture (614) and the features of the first order of intensity (158) and the shape features (12) follow. Texture features belong to the second order statistics and quantify intratumor heterogeneity by measures like correlation, dissimilarity, energy, entropy, homogeneity, and second-order. We have found that texture features are discriminative for the EGFR mutation status. This finding is in line with previous study demonstrating that EGFR(+) tumors are more heterogeneous than EGFR(-) (10).

Among the 50 highly informative features, the features of First Order Feature have the largest number, reaching 17. Meanwhile, 201 of the GLCM features are selected from 1,037 features and added to the 784 features used by the deep learning model. It is known that GLCM can calculated measures of higher order statistics including contrast and coarseness (43).

To know the reproducibility of segmentation and resulted features, we have conducted two experiments: 1) the test-retest reproducibility of 3D U-Net segmentation; 2) the reproducibility of segmentation using 3D U-Net and 3D V-Net. The intra-class correlation coefficient (ICC) has been calculated between the features obtained from two segmentations. The mean value of ICCs for 205 patients is 0.947 for the test-retest reproducibility and 0.811 for segmentations using 3D U-Net and 3D V-Net.

### Machine Learning Models Using Radiomics and Clinical Features

Machine learning models using radiomics features are the mainstream of radiomics study including EGFR mutation prediction from CT images. Our SVM model with 50 radiomics feature has presented good performance (AUC = 0.778). Velazquez et al. have achieved an AUC of 0.69 using manually extracted CT features and radiomics method (10). Gevaert et al. have also gotten good results through the decision tree model in the cohort of 186 patients (42). Lu et al. have even obtained one AUC of 0.90 for 104 patients (44). It should be noted that our cohort includes 709 LADC patients and is much higher than previous study, suggesting the higher generalizability and lower over-fitting problem.

The advantage of machine learning models using radiomics features are two aspects. First, the radiomics features are usually well-defined according to expert domain knowledge, can be understandable for observers and usually semantic. For example, the features of CT image intensity reflects the attenuation coefficient of tissues to X ray; the shape and size features characterize the tumors’ elongation, sphericity, and compactness; the texture features quantify the intratumor heterogeneity and possible necrosis (10). Moreover, many agnostic features of higher order and filtered metrics can also be captured. Second, the cohort can be small if the rule of thumb can be satisfied, i.e., each feature requires 10 patients in a binary classifier (7).

Clinical features can be predictive for EGFR mutation status by the aid of machine learning. We have used the clinical features and SVM to build one model with an AUC of 0.751 for the internal test cohort. About whether the relationship between clinical and radiomics features are complementary, our results are different with previous study. In our study, the combination of clinical and radiomics features do not increase the prediction performance. On the contrary, Velazquez et al. have presented that the fusion of clinical and radiomics features can improve prediction result (10). Li et al. have reported that the AUC increases from 0.76 to 0.79 by inclusion of the clinical features (45). The possible reason might lie on the fact that our radiomics model and clinical model have reach the high AUC separately and there is no margin for further improvement, even for the combination.

### Deep Learning Models Directly Using CT Images

Regarding the prediction of EGFR mutation status through CT images, deep learning can get better performance than machine learning of predefined engineered features. Using transfer learning of DenseNet pre-trained with 1.2 million natural images, Wang et al. have realized encouraging performance (AUC 0.81) for an independent validation cohort of 241 patients (26). Recently a deep learning model fusing CNN and long short-term memory (LSTM) has presented good prediction performance (46). Deep learning model can automatically learn multi-level features by using a neural network that are difficult to be formulized but directly related to EGFR information. However, there are some limitations for deep learning models directly trained with CT images. Most deep learning models are opaque or “black box”. Although some visualization methods such as Grad-CAM have developed, the specific meaning of the features is still difficult to be explained clearly (32). Moreover, deep learning models directly using CT images are intensively data-hungry and require a lot of calculation power and a long calculation time in training.

### Deep Learning Models Using Radiomics Features

Deep learning models using radiomics features proposed in our study have created a new strategy of building hybrid system. This strategy can utilize both the powerful capability of pattern recognition of deep learning and the good interpretability of radiomics features obtained by feature engineering. Our resulted model has exerted conformity advantage of “1+1 > 2” and achieved the higher AUC than machine learning models (0.841 versus 0.778 for the external test cohort). This advantage relies on two pillars. The first pillar is the specially designed SE-CNN. By learning the global information captured, SE layer can suppress less useful features and emphasize the informative feature. Hence, SE layer can improve the prediction performance (38). SE layer can also make the CNN model converge faster during training. Moreover, our SE-CNN is rather “shallow” compared with other traditional CNNs such as VGG, ResNet and Xception. For medical imaging applications, this kind of “shallow” CNNs usually shows better performance due to the limited training data (39).

The second pillar is the radiomics feature mapping. For machine learning models, the feature number cannot be so many for the limited patients or samples, or the over-fitting will be serious (47). SE-CNN may overcome this limit since our SE-CNN model does present serious over-fitting though the mappings with 784 discriminative features are used for the training dataset of less than 700 patients. More important is that these radiomics features are interpretable and their contributions can be ranked by classical feature selection algorithm.

In parallel, another way of build hybrid system is to apply the deep CNN as feature extractor and the machine leaning as the classifier. For example, Tang et al. have used a CNN model and SVM as feature extractor and classifier, respectively (48). Even mixed features from deep learning and feature engineering and multiple instance learning (MIL) have been used in this hybrid way (49, 50). This strategy can naturally be applied to the prediction EGFR mutation status from CT images in future research.

The CNN can learn the spatial pattern of pixels in a deeply abstract way. Actually, the features are ranked according to the importance for classification by RF method and then arranged in a determined sequence for generate a mapping in our study. We think the spatial pattern of features (or pixels) should be different between EGFR(+) and EGFR(-) and the SE-CNN can learn the pattern differences. Moreover, we have tried the mapping with different arrangements, but no significant difference is found for the predictive performance. We have tried 1D-CNN and CNN without SE and found that their performance is not as good as that of SE-CNN model.

For our method of constructing the feature mapping, the augmentation cannot be used during training the deep learning models. To alleviate the overfitting, our SE-CNN only has two convolutional layers. For VGG16 and VGG19 for comparison, we have used the pre-trained CNN with fine tuning (transfer learning). We have found that SE-CNN model can recognize EGFR mutation status with an AUC of 0.910 and 0.841 for the internal and external test cohorts, respectively. An AUC of 0.841 indicates that our SE-CNN has a reasonable generalization capability and the overfitting is not so serious.

Besides the feature mapping of 28×28 that we have selected currently, we have also tried the feature mappings of 24×24 and 32×32. While using the 24×24 mapping, AUC is 0.905 and 0.815 for the internal and external test cohorts, respectively. While using the 32×32 mapping, it is 0.901 and 0.814, respectively. It indicates that to select the feature mapping with a size of 28×28 might be reasonable.

### Limitations and Future Directions

Despite the good performance of SE-CNN model in recognition of EGFR mutation status, there are still a number of limitations in our research. First, EGFR mutations may have different results between different races, but all patients are recruited in the two large tertiary referral centers in China in our research. Therefore, the results may lack universality. Second, all patients we analyzed are with lung adenocarcinoma but no patients with other histological subtypes are involved. Third, feature engineering-based radiomics methods require precise tumor boundary annotation from image data; it takes a lot of time to process the raw data.

In future research, the data can be collected from patients with multiple races. An end-to-end pipeline including automatic tumor identification, localization, and EGFR status prediction can be developed. Integration of radiomics features, clinical features and multi-level features in deep learning models may improve the predictive performance.

## Conclusion

Utilizing radiomics feature mapping extracted from non-invasive CT images, the deep learning model of SE-CNN can precisely recognize EGFR mutation status of LADC patients. The proposed method integrates both the powerful capability of pattern recognition of deep learning and the good interpretability of radiomics features. This new strategy of building hybrid system has demonstrated superior prediction performance than both the pure deep learning and machine learning, hence can be expanded to other medical applications. The radiographic phenotype of LADC is capable of reflecting the genotype of EGFR mutation, via deep learning and radiomics method. The resulted SE-CNN model may help make decision on usage of EGFR-TKI for LADC patient in an invasive, repeatable, and low-cost way.

## Data Availability Statement

The CT images will be available upon reasonable request after approval by the Ethic Committee of The First Affiliated Hospital of Guangzhou Medical University and Shengjing Hospital of China Medical University.

## Ethics Statement

The studies involving human participants were reviewed and approved by the ethics committee of The First Affiliated Hospital of Guangzhou Medical University. Written informed consent for participation was not required for this study in accordance with the national legislation and the institutional requirements.

## Author Contributions

BZ performed experiments and analyzed the data. SQ, WQ, YY, and YG proposed the idea, made discussions, and composed the manuscript together with BZ. XP collected and analyzed the data. CL directed the algorithm development and analyzed the data. All authors contributed to the article and approved the submitted version.

## Funding

This study was supported by the National Natural Science Foundation of China (Grant number: 81671773, 61672146), the Fundamental Research Funds for the Central Universities (Grant number: N181904003, N172008008, N2024005-2), and Science and Technology Planning Project of Guangdong Province (Grant number: 2017A040405065).

## Conflict of Interest

The authors declare that the research was conducted in the absence of any commercial or financial relationships that could be construed as a potential conflict of interest.

## Supplementary Material

Supplementary Figure1 The architecture and parameter settings of 1D-CNN model.
