# Supplementary figures and images for: Deep CNN Model Using CT Radiomics Feature Mapping Recognizes EGFR Gene Mutation Status of Lung Adenocarcinoma
Source: Front Oncol. 2021 Feb 12;10:598721. doi: 10.3389/fonc.2020.598721 (PMC7907520; doi:10.3389/fonc.2020.598721)

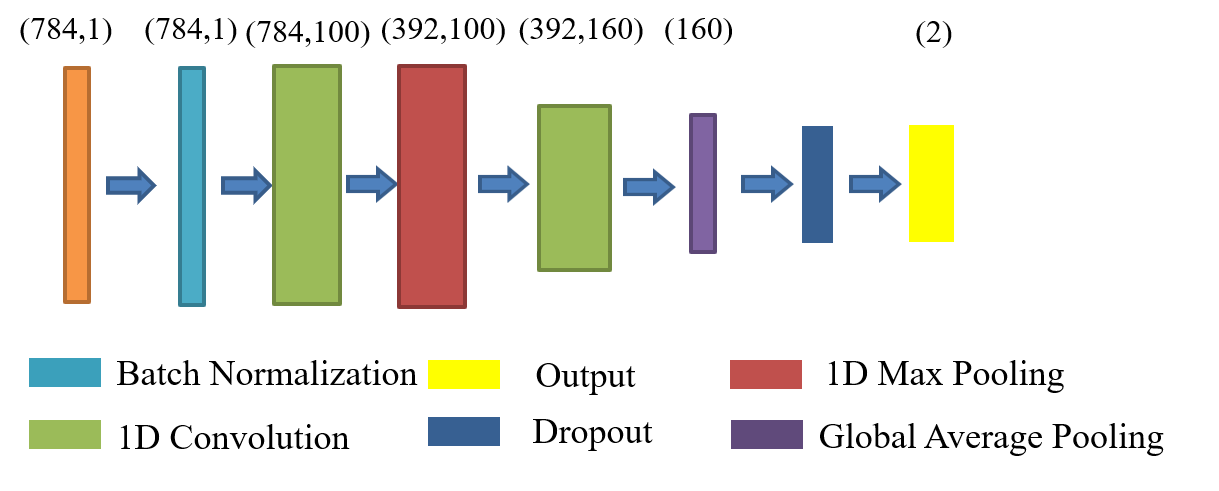

Supplement: Supplementary Figure1 — The architecture and parameter settings of 1D-CNN model. [file Image_1.png]
